# Analytical level set fabrication constraints for inverse design

**DOI:** 10.1038/s41598-019-45026-0

**Published:** 2019-06-21

**Authors:** Dries Vercruysse, Neil V. Sapra, Logan Su, Rahul Trivedi, Jelena Vučković

**Affiliations:** 1 Ginzton Laboratory, Stanford University, Stanford, California 94305 USA; 2 Departement of Physics, KULeuven, CelestijnenLaan 200 D, 3001 Heverlee, Belgium

**Keywords:** Applied optics, Silicon photonics, Nanoscale devices

## Abstract

Inverse design methods produce nanophotonic devices with arbitrary geometries that show high efficiencies as well as novel functionalities. Ensuring fabricability during optimization of these unrestricted device geometries is a major challenge for these design methods. In this work, we construct a fabrication constraint penalty function for level set geometry representations of these devices. This analytical penalty function limits both the gap size and boundary curvature of a device. We incorporate this penalty in a fully automated optical design flow using a quasi-Newton optimization method. The performance of our design method is evaluated by designing a series of waveguide demultiplexers (WDM) and mode converters with various footprints and minimum feature sizes. Finally, we design and experimentally characterize three WDMs with a 80 nm, 120 nm and 160 nm feature size.

## Introduction

Photonic design is becoming more complex and demanding as a growing number of applications are relying on nanophotonic devices. To meet this challenge, designers increasingly rely on advanced optimization techniques rather than classical photonic design approaches^1–4^. Instead of tuning relatively simple known geometries with a limited set of parameters, as is traditionally done, these new methods explore devices with completely arbitrary geometries. Capitalizing on the increased degrees of freedom, devices have been designed with extremely small footprints, high efficiencies, and novel functionalities that can not be achieved with classical methods^5–14^.

A robust design method not only needs to maximize performance, but also needs to ensure that the final device is fabricable. Various fabrication processing limits, e.g., lithography resolution or etch aspect ratio, restrict the minimum feature size that can be achieved practically. Effectively enforcing a minimum feature size on an arbitrary design geometry is challenging yet essential to produce a fully automated design algorithm. Often, optimization problems maintain fabricability by parametrizing simple geometric shapes that cannot violate the minimum feature size, such as hole arrays or pixelated structures^15,16^. This, however, restricts the geometry and thus limits the design space. For a fully arbitrary structure, feature size can be controlled by systematically projecting intermediate results on to the space of fabricable designs^3,17,18^. Such projection steps impede the use of higher order optimization methods, which decrease the optimization time and computational requirements. Another approach is to evaluate a dilated and eroded version of the device during the optimization process. Extensions of this method can allow for fabrication robustness^9,19,20^, or a length scale constraint^21,22^.

In this work, we introduce an analytical fabrication constraint for arbitrary geometries defined by level set functions. This constraint has the advantage that it can be added to the objective as a penalty term, allowing for simultaneous optimization of performance and fabricability with quasi-Newton methods. This is in contrast to previous work where fabrication constraints were enforced through systematic corrections to the device geometry^18^. Expanding on the design methodology established in our group^2,6,11,18^, we present a completely automated optimization process which implements fabricabilty through this penalty term. The performance of this optimization process is evaluated for wavelength demultiplexers (WDMs) and mode converters with varied footprints and minimum feature sizes. Finally, we employ our method to design three WDMs with different minimum feature sizes, which are fabricated and experimentally characterized.

## Level Set Fabrication Constraint

**Figure 1:**
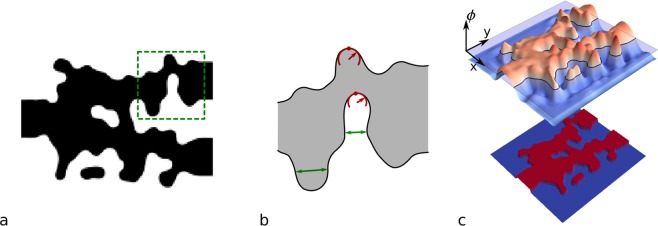
Level set representation: (**a**) example of a discrete device structure, (**b**) illustration of feature sizes in the green region in panel (a). The curvature is indicated in red, and the gap is shown in green, (**c**) level set parametrization of the example structure in panel (a) with level set thresholding depicted.

To facilitate optimization, a device geometry is often represented by binary pixels on a grid that matches the simulation mesh or the minimum fabricable feature size^7,16,23^. This Manhattan representation, however, limits the design space. Preferably, the design border should be able to move continuously. This can be achieved by directly parameterizing the device boundary as a polygon^24^, or indirectly by using a level set function, as is done in this work. A level set function is a continuous function that defines material where the function is positive, and etch where the function is negative, thereby setting the boundary to be the zero-crossing^25^. An example of a 2D level set function can be seen in Fig. 1(c).

For the device to be considered fabricable, the geometry needs to adhere to a target minimum feature size, *d*, which the fabrication process can resolve. This requires, firstly, that there are no gaps smaller than the minimum feature size, and secondly, that the radius of curvature is larger than half the minimum feature size (Fig. 1(b)). Without enforcing these constraints during the optimization, the final designs typically have small features that can be difficult to fabricate, e.g., features smaller than 80nm (Suppl. Information). To ensure fabrication requirements, we introduce two level set specific constraints to the optimization problem: a minimal gap and radius of curvature constraint.

**Figure 2:**
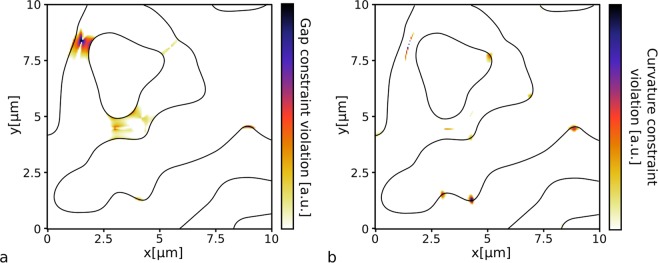
Fabrication constraints: violations of a 1 μm gap constraint (**a**) and curvature constraint (**b**) are indicated for a randomly generated level set function. The black line indicates the zero-contour of the level set function.

The minimal gap size is enforced by the constraint:

1 $$|{\varphi }_{vv}(x,y;p)| < {(\frac{\pi }{d})}^{2}\cdot |\varphi (x,y;p)|+\beta \cdot \frac{\pi }{d}\cdot {\varphi }_{v}(x,y;p),$$

where *p* is a vector describing the parametrization; $\varphi (x,y;p)$ is the level set function; ${\varphi }_{v}(x,y;p)$ and ${\varphi }_{vv}(x,y;p)$ are the first and second derivatives, respectively, in the gradient direction^26^; and $\beta  > 0$ is a constant. This constraint can be understood intuitively when ignoring the second term. Since the second derivative indicates how quickly the level set function bends back towards the zero-plane, the feature size is controlled by limiting the second derivative based on the value of the function at that point. For a 1D level set function, this constraint is tight for a sinusoidal function with a periodicity 2*d*, which corresponds to a grating with feature size *d*. The additional term, $\beta \cdot {\varphi }_{v}(x,y;p)$, is added to relax the constraint near the zero-plane, so that the second derivative would not have to be exactly zero (for numerical reasons). *β* is typically set to $\frac{1}{3}$. An example of where this constraint is violated can be seen in Fig. 2(a).

The minimum radius of curvature, *r*, is enforced by the constraint:

2 $$r(x,y;p)={(\nabla \cdot (\frac{\nabla \varphi (x,y;p)}{|\nabla \varphi (x,y;p)|}))}^{-1} > |\arctan (\frac{{\varphi }_{v}(x,y;p)}{\varphi (x,y;p)})\cdot \frac{d}{\pi }|.$$

Although the curvature is only relevant at the device boundary ($\varphi =0$), the constraint is evaluated over the entire design region. The constraint is formulated this way because only penalizing curvature at grid points near the boundary results in a highly non-differentiable penalty function, which hinders the optimization process. At points where $\varphi (x,y;p)=0$, the arctan-term in Equation (2) will be $\frac{\pi }{2}$, in which case the constraint becomes $r(p) > \frac{\pi }{2}\cdot \frac{d}{\pi }=\frac{d}{2}$. At the extrema, where ${\varphi }_{v}(x,y;p)=0$ and the radius of curvature is 0, the arctan-term will be 0 effectively removing the constraint. As such Equation (2) constrains the radius of curvature to $\frac{d}{2}$ at the device border (i.e., at the level set zero-plane) and relaxes it away from the device boundary. Figure 2(b) shows where this constraint is violated in an example structure.

Both constraints can be combined in a penalty function:

3 $$\begin{array}{rcl}{f}_{fab}(p) & = & {\iint }_{A}\,dxdyR(\frac{|{\varphi }_{vv}(x,y;p)|}{\frac{\pi }{d}|\varphi (x,y;p)|+\beta {\varphi }_{v}(x,y;p)}-\frac{\pi }{d})\\  &  & +\,\zeta \cdot {\iint }_{A}\,dxdyR(|\frac{1}{r}\arctan (\frac{{\varphi }_{v}(x,y;p)}{\varphi (x,y;p)})|-\frac{\pi }{d}),\end{array}$$

where *R* is the ramp function, $R(x)=\,{\max }(x,0)$. The penalty weight, $\zeta$, sets the relative importance between the gap and curvature penalty terms, which was set to two in the present implementation. If both constraints 1 and 2 are met, the penalty function will be zero. We observe that optimized devices that meet the constraints do not meet the target minimum feature size, *d*, set in these equations, but tend to be consistently smaller. Therefore, during optimization, we set *d* to be 15% higher than the target feature size.

## Inverse Design

An optical design problem with the fabrication penalty in Equation (3) can be formulated as:

4 $$\begin{array}{cc}\mathop{{\rm{m}}{\rm{i}}{\rm{n}}{\rm{i}}{\rm{m}}{\rm{i}}{\rm{z}}{\rm{e}}}\limits_{p,{E}_{1},\ldots ,{E}_{n}} & {f}_{EM}(p,{E}_{1},\ldots {E}_{n})+\tau \cdot {f}_{fab}(p)\\ {\rm{s}}{\rm{u}}{\rm{b}}{\rm{j}}{\rm{e}}{\rm{c}}{\rm{t}}\,{\rm{t}}{\rm{o}} & {\rm{\nabla }}\times \frac{1}{\mu }{\rm{\nabla }}\times {E}_{i}-{\omega }_{i}^{2}\varepsilon (p){E}_{i}=-\,j{\omega }_{i}{J}_{i},\,i=1,\ldots ,n,\end{array}$$

where *f*_*EM*_ is a figure of merit for the optical performance as a function of the fields *E*_*i*_ and the parametrization vector, *p*. This optimization problem is subject to Maxwell equations, where ${\omega }_{i}$ and *J*_*i*_ are the angular frequency and current sources of mode *i*, respectively, and $\varepsilon (p)$ is permittivity as a function of the parametrization.

**Figure 3:**
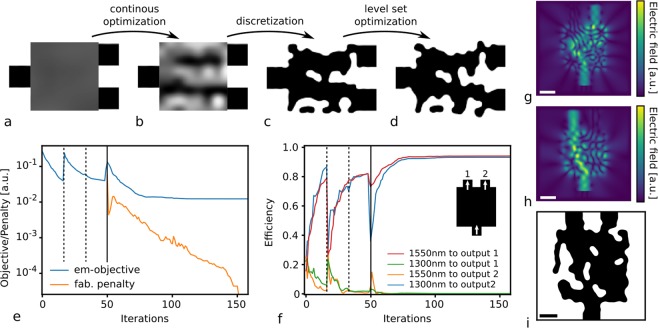
Inverse design method (**a**–**d**): (**a**) random initial condition for a waveguide demultiplexer, (**b**) structure after optimization in continuous stage, (**c**) structure after discretization step, (**d**) structure after optimization with a level set parametrization and fabrication constraints. WDM optimization with fabrication constraints (**e**–**i**): (**e**) EM-objective (*f*_*EM*_) and fabrication penalty (*f*_*fab*_) at every iteration, (**f**) coupling efficiency at every iteration. The dotted vertical line in a and b indicates a change in the sigmoid function slope (Suppl. Information). The full vertical line indicates the continuous-to-discretization step. (**g**,**h**) Electric field intensity of the final structure at 1300 nm and 1550 nm wavelength, respectively, and (**i**) final structure. The scale bar in (**g**–**i**) is 0.5 μm.

Our inverse design method optimizes the optical problem in Equation (4) in three stages: a continuous optimization stage, where the permittivity of the design region can take any value in between the waveguide and the cladding; a discretization stage, where the continuous result is discretized; and a discrete optimization stage, where the level set representation of the device is optimized with fabrication constraints (Fig. 3a–d)^2,6,11^.

In the first stage (continuous) the parametrization is not a level set function, and as such we omit the penalty term from Equation (4). Yet, the feature size in this stage still needs to be controlled, since small features at the end of the continuous optimization will result in a poor starting condition for the discrete stage. We, therefore, use a coarser grid for the parameterization and interpolate onto the finer simulation grid using cubic interpolation (Suppl. Information). This coarse grid has a pitch of 1.75 times the required minimum feature size. In addition, we apply a sigmoid function over the interpolated result, in order to make the continuous structure more discrete (Suppl. Information). An example of the permittivity distribution for a WDM at the end of a continuous stage can be seen in Fig. 3(b).

The non-fabricable structure is subsequently discretized as illustrated in Fig. 3(b,c) to form the initial guess for the second optimization stage (discrete). Here, the device is strictly composed of regions with the waveguide permittivity and regions with the cladding permittivity, i.e., etched regions. To discretize, we fit a level set parametrization to the continuous optimization result, taking *f*_*fab*_(*p*) into account to assure fabricability. The fitted result is subsequently used as a starting condition for the discrete optimization stage. In the final discrete stage, we solve the optimization problem shown in Equation (4) (Fig. 3(c,d)). The weight factor $\tau$ is increased ten times over the optimization process, and each sub-optimization is solved using the quasi-Newton method L-BFGS-B in order to yield fast convergence.

The penalty term in Equation (4) introduces a challenge to solve the problem directly on a fine simulation grid. Rather than directly parameterizing on the simulation grid, we again use a coarse grid and interpolate on the finer simulation grid to smoothen the optimization landscape (Suppl. Information).

## Results and Discussion

### WDM with fabrication constraints

The optimization results for a 1300 nm/1550 nm WDM is shown in Fig. 3(e–i). The device was optimized in 2D with a 2.5 μm × 2.5 μm design area, the refractive index of the waveguide and surrounding cladding is 3.48 and 1.44, respectively, and the target minimum feature size, *d*, was set to 120 nm. Figure 3(e) shows the electromagnetic (EM) objective and penalty function during the optimization process. The continuous optimization stage takes 48 iterations, during which the sigmoid function slope, *k*, is changed twice. This change results in a large increase in the EM-objective at the 16^th^ iteration and a small increase at the 32^nd^ iteration. After the continuous stage, the structures is discretized, which again causes a performance decrease. At this point, the optimization relies on a level set parametrization and the problem in Equation (4) is solved. The penalty function for the gap and the curvature in Equation (3) is shown in Fig. 3(a) by the orange curve. Over the discrete optimization stage from iteration 49 to 153, the EM-objective is reduced to 1.5 × 10^−3^, and the fabrication penalty function reaches 0. The final structure (Fig. 3(i)) has an efficiency of 93% and 92% for 1300 nm and 1550 nm, respectively (Fig. 3(f)). The field profiles for both wavelengths can be seen in Fig. 3(g,h). With the penalty function reaching 0, both fabrication constraints are completely met. The device has a minimum gap size of 121.2 nm and a minimum radius of curvature of 63.1 nm.

### Minimum feature size vs device footprint

**Figure 4:**
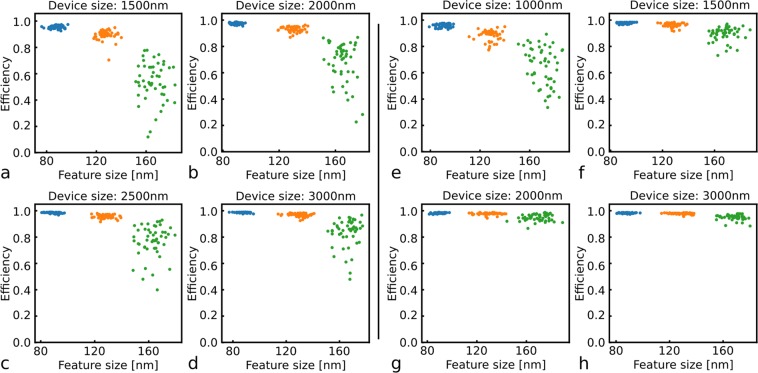
Parameter sweep: (**a**–**d**) efficiency of WDMs and (**e**–**h**) efficiency of TE0-to-TE1 mode converters with different footprints and different minimum feature size. Every sweep uses 50 random starting conditions.

The inverse design method was evaluated by optimizing a series of optical devices with different device parameters. WDMs for 1300 nm/1550 nm were optimized for footprints ranging from 1.5 μm × 1.5 μm to 3 μm × 3 μm and target minimum feature sizes of 80 nm, 120 nm and 160 nm. For each device configuration, we optimized 50 devices starting with different random initial conditions. Of the two optimized wavelengths, the lowest efficiency value is given as the device efficiency in Fig. 4(a–d). The x-axis of these figures shows the minimum feature size of the optimized device, i.e., the minimum of the gap sizes and curvature diameters found in the device geometry (Suppl. Information). In all four figures, the device minimum feature size lies around the target minimum feature size. A small number of the designs still violate the requirement, yet this could be improved by setting the constraint slightly higher, or by increasing the pitch of the coarse grid. Alternatively, when increasing the penalty factor, $\tau$, one could also perform an optimization on the penalty term only to obtain a fabricable structure as a starting condition. Several trends regarding the minimum feature size and the device footprint are visible. For each footprint, the maximum efficiency decreases as the minimum feature size increases. For example, 1.5 μm × 1.5 μm WDMs with an 80 nm feature size can reach efficiencies of 96.6%, but the highest efficiency for the 160 nm feature size is 77.1%. In addition, the spread on the efficiency also becomes larger as the target feature size increases. While all 1.5 μm × 1.5 μm WDMs with a minimum feature size of 80 nm reaches an efficiency between 91.8% and 96.6%, the efficiencies of devices with a 160 nm feature size spans from 11.2% to 77.1%. These trends appear consistently over all the device footprints. Between different device footprints, maximum efficiency increases as the footprint increases. For the 160 nm feature size, the maximum efficiency for a 1.5 μm × 1.5 μm device is 77.1%, while a 3 μm × 3 μm WDM can reach almost 96.7%. Additional optimization metrics, such as average iteration counts and computation time, can be found in the Supplementary Information.

A similar parameter sweep was done for a TE0-to-TE1 mode converter. The mode conversion efficiencies for 50 optimizations with different footprints and minimum feature sizes are shown in Fig. 4(e–h). The same trends as for the WDM can be observed. The effect of the device footprint on the efficiency spread is nevertheless more pronounced. For example, the 160 nm minimum feature size has an efficiency range from 33.6–89.3% for the 1 μm × 1 μm device, yet for the 3 μm × 3 μm device range is already reduced to 88.4–97.8%. The optimization landscape of the mode converter presumably has higher performing local minima to which the device can converge, making it more likely to find high-efficiency devices as compared to the WDM problem.

### 3D WDM designs

**Figure 5:**
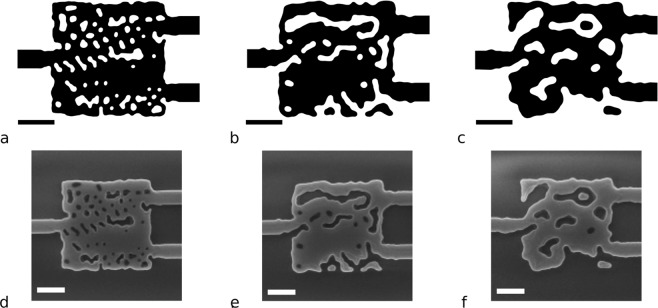
3D designs and SEM micrographs of WDMs with a 80 nm(**a**,**d**), 120 nm(**b**,**e**) and 160 nm(**c**,**f**) minimum feature size. The scale bar in both the design and SEM images are 1 μm.

Three-dimensional WDM’s with a footprint of 3 μm × 3 μm were developed in 220 nm thick SOI with top oxide cladding for 1300 nm/1550 nm wavelengths (Fig. 5). We fabricated a set of devices whose minimum feature size, *d*, was set to 80nm, 120nm and 160nm. Table 1 summarizes the efficiencies and the fabrication feature sizes of all three devices. These devices adhere to both fabrication constraints, and as expected, devices with a smaller minimum feature size have a higher efficiency (Full spectra can be found in the Suppl. Information). The experimental measurement follows the trend seen in simulations, yet show an overall lower efficiency which can be attributed to fabrication imperfections.

**Table 1:** 3D WDM results for different minimum feature sizes.

Feature size (*d*)	1300 nm efficiency FDFD/experiment*	1550 nm efficiency FDFD/experiment*	Curvature diameter	Gap size
80 nm	95.0%/79.0%	94.1%/59.0%	82.0 nm	81.7 nm
120 nm	71.9%/48.6%	72.4%/39.7%	124.2 nm	121.8 nm
160 nm	52.7%/27.4%	60.0%/39.8%	163.8 nm	161.1 nm

*Experimental results take a blue shift into account (Suppl. Information).

## Conclusion

We have developed analytical constraints that limit the gap size and curvature of a structure defined by a level set function. These constraints were incorporated in a fully automated inverse design method in the form of a penalty function. Using this new penalty function, we optimized a series of 2D WDMs and mode converters with varied footprints and minimum feature sizes. Analysis of the feature size of a series of devices that are optimized with certain target feature size, shows a distribution with a lower bound around this target. In addition, the optimization series demonstrate that the spread in the final efficiency of optimized devices increases as the fabrication constraint increases. To improve optimization with large minimum feature sizes, future work might focus on improving initial conditions and on early termination to reduce the computational load of sweeping initial conditions. The validity of this method was demonstrated through simulation in both 2D and 3D, as well as experimental confirmation of device performance. The robustness and flexibility afforded by the presented design approach illustrates the maturity of arbitrary geometry nanophotonic design for widespread use in the integrated photonics community.

## Supplementary information

Supplementary Information
