# Supplementary material for: Analytical level set fabrication constraints for inverse design
Source: Sci Rep. 2019 Jun 21;9:8999. doi: 10.1038/s41598-019-45026-0 (PMC6588594; doi:10.1038/s41598-019-45026-0)
Supplement: Supplementary file 1 — Supplementary Information [file 41598_2019_45026_MOESM1_ESM.pdf]

Supplementary Information: Analytical level set fabrication constraints for inverse design

Dries Vercruysse[1,2], Neil V. Sapra[1], Logan Su[1], Rahul Trivedi[1], and Jelena Vučković[1]

[1]Ginzton Laboratory, Stanford University, Stanford, California 94305, USA
[2]Departement of Physics, KULeuven, CelestijnenLaan 200 D, 3001 Heverlee, Belgium
[*]vcruysse@stanford.edu

1 Final feature size for unconstrained optimization

Figure 1(a-d) shows the histograms of the minimum feature size for four series of 50 TE0-to-TE1 mode converters optimized without fabrication constraint. All devices reached a > 0.985 efficiency. As the size of the device increases the prevalence of smaller features increases as well. In all four series the minimum features size is well below 80 nm, which we typically use as a fabrication limit.

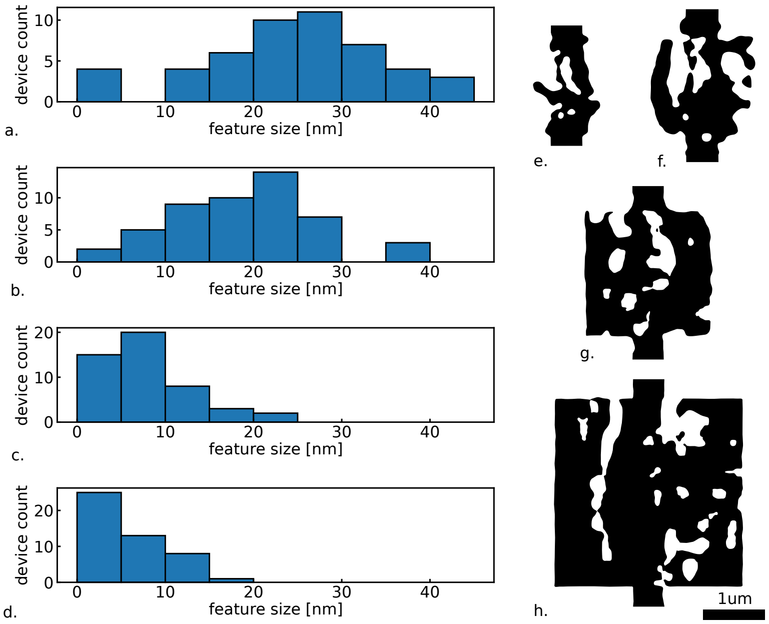

Figure 1. Optimization results of TE0-to-TE1 mode converters without fabrication constraints: (a-d) Final feature size distribution for devices with a 1000 nm × 1000 nm, 1500 nm × 1500 nm, 2000 nm × 2000 nm and 3000 nm × 3000 nm device size, respectively. (e-f) Example device out of the distributions shown in panel a-d, respectively.

2 Experimental results

The efficiency of the WDM is measured using the photonic circuit schematically depicted in Figure 2(a). Light from a supercontinuum source (Fianium SC400-4) is coupled in by the input grating on the left, collected at the output gratings and send to an optical spectral analyzer. The circuit splits the input light in two branches by a multimode interference coupler (MMI). One branch of the MMI connects to the WDM which connects the two output gratings. The other branch connects to the reference output grating. The efficiency spectra shown in panels b-c of Figure 2 are the ratio of the WDM output grating power and reference grating power. Since no grating can efficiently span the entire spectral range of interest, the circuit was repeated with different gratings. The three different gratings span from 1200-1340nm, 1340nm-1450nm and 1450-1600nm. The dashed vertical lines indicate the transitions between the gratings.

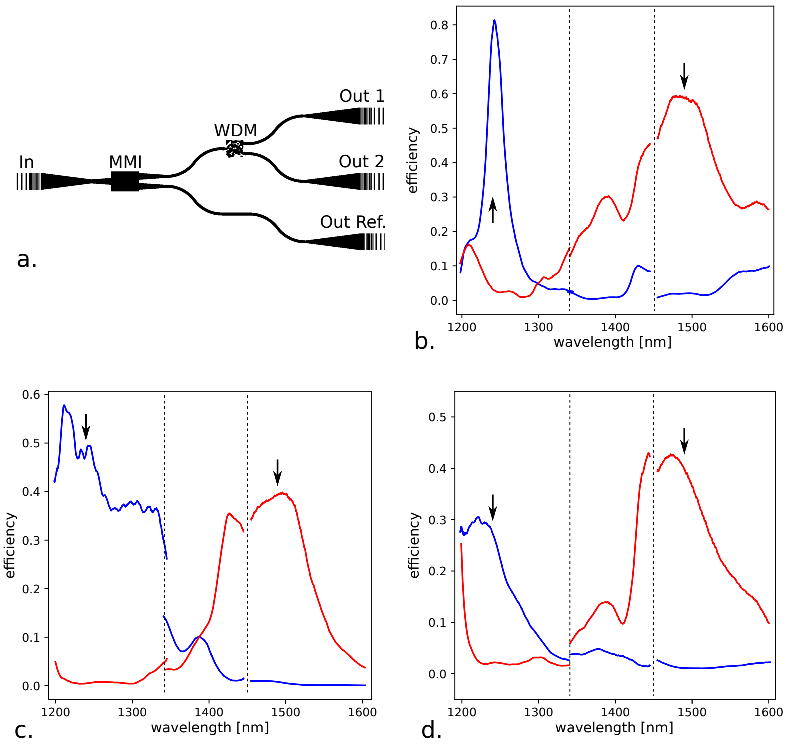

Figure 2. Experimental results: (a) schematic depiction of the measurement circuit, (b-d) spectra for the WDM designed with 80nm, 120nm and 160nm feature size, respectively. The arrows indicate the design frequency taking into account a blue shift.

3 Optimization time

After the optimization series shown in the main manuscript, we performed an additional optimization series of 10 devices to map out the computational cost of our algorithm. The optimizations were done for 2D-devices. Average iterations and function evaluation counts are shown in table 1 and 2. Time to evaluate the optimization objective function and the fabrication constraint penalty function and its gradients are shown in table 3. The optimization for each device ran on a single Intel(R) Xeon(R) CPU @ 2.20GHz and required 1 GB of RAM.

Table 1. Average and standard deviation of the L-BFGS-B iterations and function evaluation needed to optimize a TE0-to-TE1 mode converter.

design feature size	device size	average L-BFGS-B iterations	average function evaluations
80 nm	1000 nm × 1000 nm	94.1 ± 11.1	250.8 ± 48.3
80 nm	1500 nm × 1500 nm	94.2 ± 12.3	216.2 ± 43.3
80 nm	2000 nm × 2000 nm	83.9 ± 5.3	192.7 ± 30.3
80 nm	3000 nm × 3000 nm	86.2 ± 10.7	193.9 ± 59.3
120 nm	1000 nm × 1000 nm	125.78 ± 20.1	372.9 ± 45.0
120 nm	1500 nm × 1500 nm	100.2 ± 23.4	300.8 ± 110.9
120 nm	2000 nm × 2000 nm	83.6 ± 15.6	206.5 ± 60.7
120 nm	3000 nm × 3000 nm	109.0 ± 36.2	365.5 ± 260.2
160 nm	1000 nm × 1000 nm	99.8 ± 27.9	376.6 ± 137.2
160 nm	1500 nm × 1500 nm	97.0 ± 17.2	303.6 ± 78.4
160 nm	2000 nm × 2000 nm	103.8 ± 31.3	359.4 ± 128.7
160 nm	3000 nm × 3000 nm	90.5 ± 17.3	332.8 ± 109.2

Table 2. Average and standard deviation of the L-BFGS-B iterations and function evaluation needed to optimize a WDM.

design feature size	device size	average L-BFGS-B iterations	average function evaluations
80 nm	1000 nm × 1000 nm	202.3 ± 26.9	423.9 ± 92.5
80 nm	1500 nm × 1500 nm	164.4 ± 22.6	358.5 ± 95.2
80 nm	2000 nm × 2000 nm	142.4 ± 14.9	269.0 ± 72.1
80 nm	3000 nm × 3000 nm	128.0 ± 28.5	249.8 ± 86.7
120 nm	1000 nm × 1000 nm	255.8 ± 45.2	546.99 ± 104.3
120 nm	1500 nm × 1500 nm	223.4 ± 47.7	552.3 ± 191.3
120 nm	2000 nm × 2000 nm	206.0 ± 51.0	454.4 ± 123.1
120 nm	3000 nm × 3000 nm	162.8 ± 36.3	386.5 ± 151.8
160 nm	1000 nm × 1000 nm	169.6 ± 60.5	565.8 ± 270.0
160 nm	1500 nm × 1500 nm	182.7 ± 72.6	628.1 ± 196.1
160 nm	2000 nm × 2000 nm	186.6 ± 75.1	629.6 ± 282.6
160 nm	3000 nm × 3000 nm	183.9 ± 51.0	596.3 ± 237.7

Table 3. Average time to evaluate the EM term and penalty term of the objective function, t_f and its gradients, t_{grad}, for different device sizes and target feature sizes. The values were obtained for the optimization of TE0-to-TE1 mode converter.

design feature size	device size	average evaluation time for EM term	average evaluation time for penalty term
80 nm	1000 nm × 1000 nm	$t_f = 0.1202$ s, $t_{grad} = 0.1291$ s	$t_f = 0.0202$ s, $t_{grad} = 0.3579$ s
80 nm	1500 nm × 1500 nm	$t_f = 0.1765$ s, $t_{grad} = 0.1882$ s	$t_f = 0.0381$ s, $t_{grad} = 0.7582$ s
80 nm	2000 nm × 2000 nm	$t_f = 0.2057$ s, $t_{grad} = 0.2203$ s	$t_f = 0.048\,77$ s, $t_{grad} = 0.9628$ s
80 nm	3000 nm × 3000 nm	$t_f = 0.3656$ s, $t_{grad} = 0.3945$ s	$t_f = 0.1005$ s, $t_{grad} = 2.3823$ s
120 nm	1000 nm × 1000 nm	$t_f = 0.1626$ s, $t_{grad} = 0.1731$ s	$t_f = 0.0125$ s, $t_{grad} = 0.2131$ s
120 nm	1500 nm × 1500 nm	$t_f = 0.2196$ s, $t_{grad} = 0.2324$ s	$t_f = 0.0224$ s, $t_{grad} = 0.3943$ s
120 nm	2000 nm × 2000 nm	$t_f = 0.2427$ s, $t_{grad} = 0.2655$ s	$t_f = 0.0290$ s, $t_{grad} = 0.5114$ s
120 nm	3000 nm × 3000 nm	$t_f = 0.4359$ s, $t_{grad} = 0.4736$ s	$t_f = 0.0518$ s, $t_{grad} = 1.047$ s
160 nm	1000 nm × 1000 nm	$t_f = 0.2044$ s, $t_{grad} = 0.2131$ s	$t_f = 0.0103$ s, $t_{grad} = 0.1515$ s
160 nm	1500 nm × 1500 nm	$t_f = 0.2522$ s, $t_{grad} = 0.2648$ s	$t_f = 0.0136$ s, $t_{grad} = 0.2499$ s
160 nm	2000 nm × 2000 nm	$t_f = 0.2998$ s, $t_{grad} = 0.3198$ s	$t_f = 0.0186$ s, $t_{grad} = 0.3199$ s
160 nm	3000 nm × 3000 nm	$t_f = 0.5374$ s, $t_{grad} = 0.5811$ s	$t_f = 0.0331$ s, $t_{grad} = 0.5679$ s

4 Interpolation

During both the continuous and discrete stage, the device geometry is parametrized on a coarse grid. In the continuous stage, each coarse grid point defines a single function value and the finer simulation grid is interpolated using a bicubic interpolation on this coarse grid. For the discrete stage, in addition to the level set function value, the first derivative in both axis and the mixed second-order derivative are also part of the parametrization at every point, as illustrated in Figure 3. The simulation grid values in between four coarse grid points are calculated as points on the following interpolation surface:

$$p(x,y) = \sum_{i=0}^{3} \sum_{j=0}^{3} a_{ij} x^i y^j, \tag{1}$$

where the a_{ij} values are calculated based on the corner point's function and derivative values, similar as in bicubic interpolation. This parametrization provides additional degrees of freedom that help in matching the fabrication constraints.

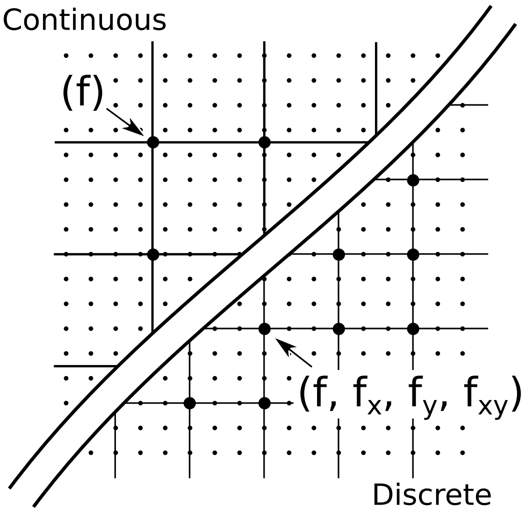

Figure 3. Illustration of the different parametrization grids used during the continuous and discrete stage. Large dots indicate parametrization points. The small dots represent the simulation mesh.

5 Continuous optimization

By interpolating on a coarse grid in the continuous stage, the device permittivity will be highly smoothened, as illustrated in Figure 4(a). This typically limits the device efficiency and results in poor initial conditions for the discrete stage after discretization. We, therefore, apply a sigmoid function on our interpolated parametrization:

$$\sigma(x) = \frac{1}{1+e^{-k\cdot(2x-1)}}, \tag{2}$$

where k is a constant that sets the slope of the sigmoid function. (figure 4(d))

Figure 4(a-c) shows the optimization result after subsequently optimizing with a $k = 4$, $k = 6$ and $k = 10$. Although applying the sigmoid function does not fully discretize the device, it does result in a device that is closer to discrete. As such, it provides better initial conditions for the discrete optimization stage.

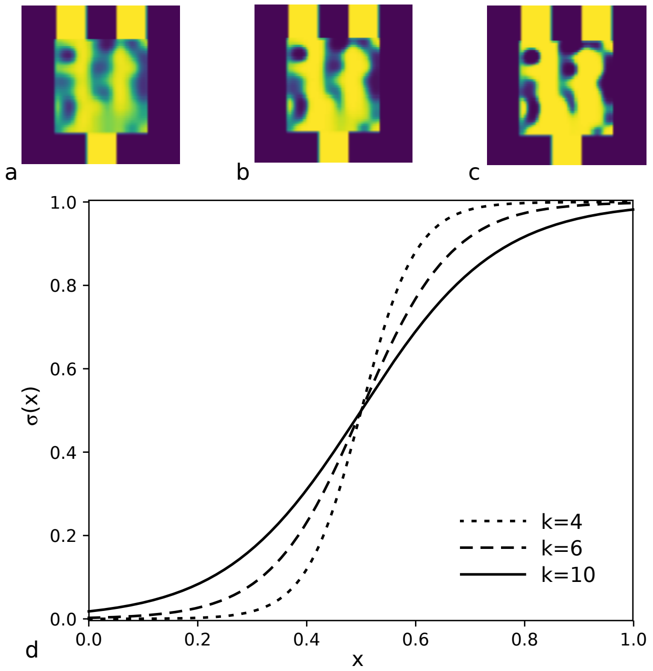

Figure 4. Sigmoid function applied during continuous optimization: (a-c) Inverse designed WDMs with $k = 4$, 6 and 10, respectively. (d) Sigmoid function with the different k values.

6 Feature Size Evaluation

The minimum feature size of the final geometry is the minimum of the smallest gap and smallest curvature diameter found in the geometry.

In order to test whether or not the target gap size is violated, we consider two points on a line perpendicular to the device boundary at the target gap distance from the boundary, as illustrated in Figure 5(a). If the level set function evaluation at the blue/red dot is positive/negative we consider the gap constraint met. This test is done at every point of the polygon that describes the device boundary (Figure 5(a)). To find the minimum gap size, we sweep the test gap size until we find a gap size that violates the constraint.

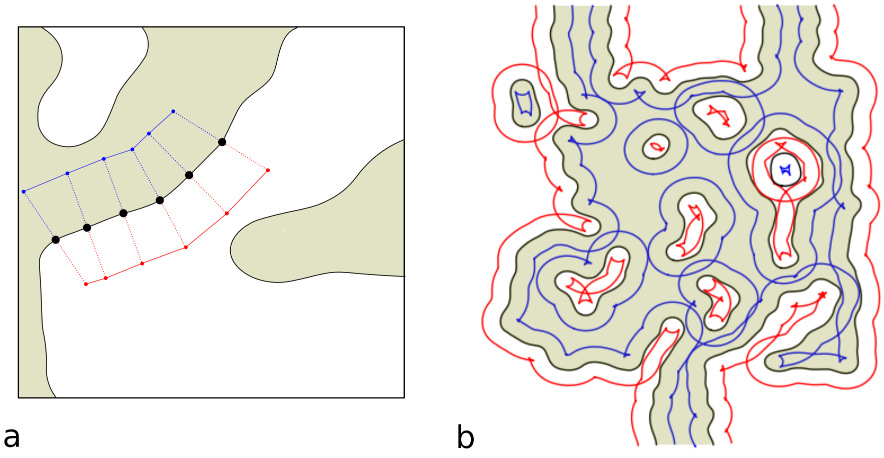

Figure 5. Gap evaluation: (a) illustration of the gap evaluation along a device boundary, (b) gap evaluation on WDM optimized with d=160nm.
